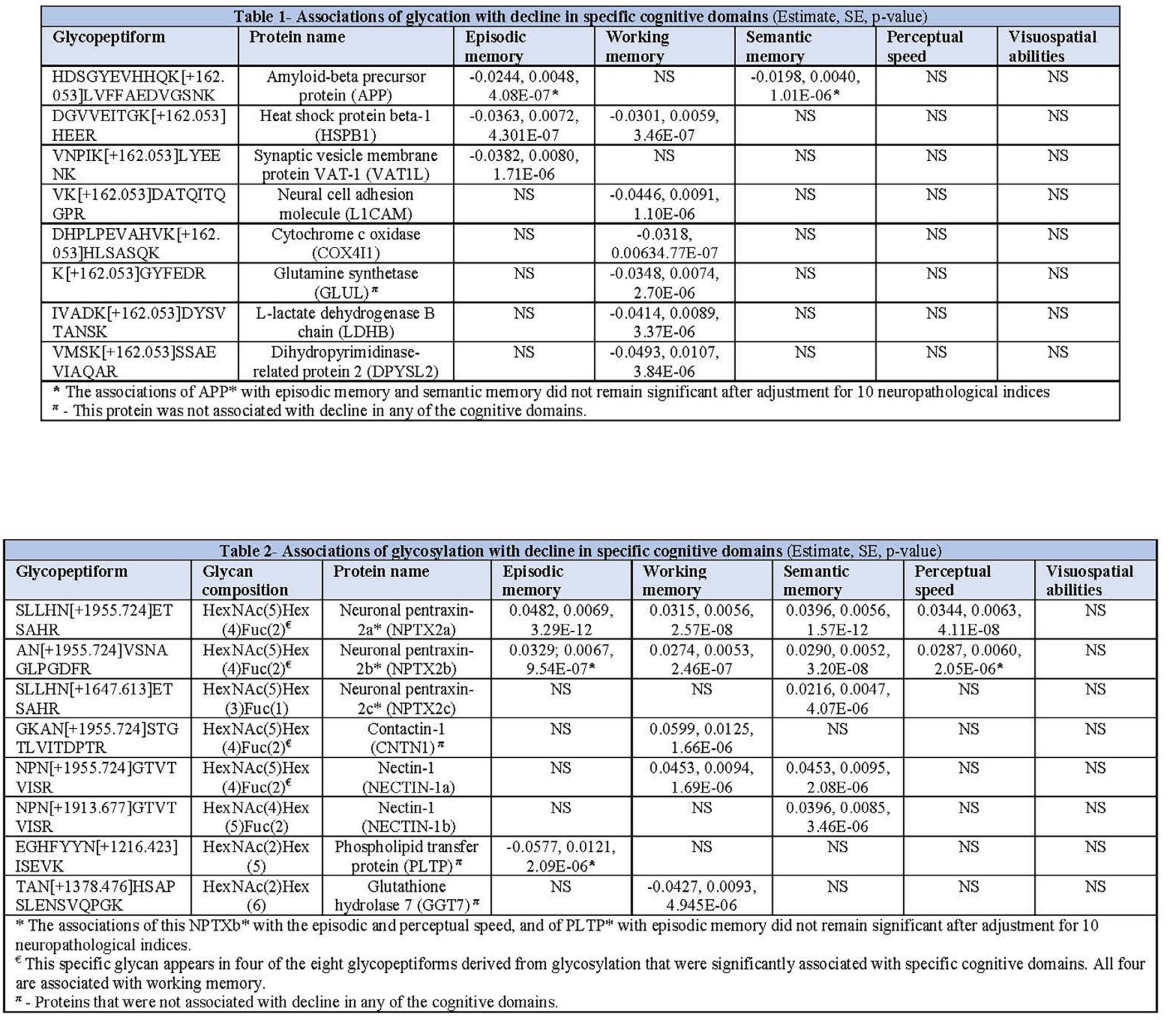# Glycoproteome‐wide analyses in the dorsolateral prefrontal cortex identified glycopeptiforms associated with cognitive decline in specific cognitive domains

**DOI:** 10.1002/alz.092943

**Published:** 2025-01-09

**Authors:** Michal S Beeri, Yishai Levin, David Morgenstern, Nili Tikotzki, Hila Levy, Itzik Cooper, Hans‐Ulrich Klein, David A. Bennett, Lei Yu, Aron S Buchman

**Affiliations:** ^1^ Herbert and Jackeline Krieger Klein Alzheimer’s Research Center, Rutgers Biomedical and Health Sciences, Newark, NJ USA; ^2^ Weizmann Institute of Science, Rehovot, Israel Israel; ^3^ Ben Gurion University, Beeri Sheva, Israel Israel; ^4^ The Joseph Sagol Neuroscience Center, Sheba Medical Center, Ramat Gan, Israel Israel; ^5^ Columbia University, New York, NY USA; ^6^ Rush University, Chicago, IL USA; ^7^ Rush Alzheimer’s Disease Center, Rush University Medical Center, Chicago, IL USA

## Abstract

**Background:**

Molecular omics studies of aging brains have identified genes and proteins related to cognitive decline. Identifying proteins that are linked to specific cognitive abilities has potential to catalyze the development of targeted therapies for specific cognitive deficits. Posttranslational modifications of proteins with glycans modify protein function. Here, we identified glycopeptiforms associated with decline in specific cognitive domains.

**Method:**

We studied brains of 366 older decedents from the Religious Orders Study and Rush Memory and Aging Project (ROSMAP) with annual cognitive testing, postmortem indices of ten AD/ADRD pathologies and proteome‐wide data, from dorsal lateral prefrontal cortex (DLPFC). We quantified 11,012 glycopeptiforms from DLPFC using liquid chromatography with tandem mass spectrometry, which is able to identify both glycated and glycosylated glycopeptiforms, two distinct molecular processes. We employed linear mixed‐effects models controlling for age, sex and education to identify glycopeptiforms associated with decline in five cognitive domains (episodic memory, semantic memory, working memory, perceptual speed and visuospatial abilities) correcting for multiple comparisons (p<5×10‐6). In further analyses, we adjusted for 10 neuropathologies and for the constituent proteins of the glycopeptiforms.

**Result:**

We found eight glycated (**Table 1**) and eight glycosylated glycopeptiforms (**Table 2**) that were significantly associated with cognitive decline. Glycated glycopeptiformswere associated primarily with decline in working memory. In contrast, glycosylated glycopeptiforms were associated with all cognitive domains except for visuospatial abilities. Higher levels of glycated glycopeptiforms were always related to faster cognitive decline while higher levels of glycosylated glycopeptiforms were primarily associated with slower cognitive decline. Three glycopeptiforms identified weresituated on the protein neuronal pentraxin 2, linked to AD. The HexNAc(5)Hex(4)Fuc(2) glycan composition appeared in half of the significant glycosylated glycopeptiforms while only in <0.05% of the 11,000 glycopeptiforms, suggesting its specific involvement in cognition. Adjustment for 10 neuropathologies attenuated 3 of the 25 significant associations. Adjustment for the constituent proteins did not alter any of the results suggesting that the post‐translational modification itself may have a role in cognitive decline.

**Conclusion:**

Glycopeptiforms in aging brains may contribute to decline in distinct cognitive abilities. Further drug discovery targeting these glycopeptiforms may lead to therapies that prevent specific cognitive deficits.